# On Nyctalopia, or Night-Blindness

**Published:** 1816-11

**Authors:** 


*For the London Medical and Physical Journal.*

### *On Nyctalopia, or Night-Blindness;* by J. W.

THIS disease, which is very common among seaman, as well as others, in warm climates, consists in a perfectly correct sight during the day-time, but, as soon as the sun begins to set, a total blindness takes place.

When it occurs in warm climates, it is owing to a want of tone in the retina and optic nerve; for, being accustomed, during the day, to the high stimulus of the power, light, and heat of the reflected rays of the sun, the excitability becomes so exhausted from it, that the weaker afforded by the night is incapable of making the slightest impression, and a temporary blindness is the consequence.

I have been informed, by very good authority, that Nyctalopia also occurs in situations opposite to the above-described—as in cold wet weather, when a great many people, with their wet clothes on, are crowded together, with little ventilation, and without fires, as on-board of ship, in long-continued gales of wind, in the winter season.—This I have never seen myself.

A curious circumstance attending the complaint is, that when a lighted candle is placed before the eye, the pupil, instead of contracting, dilates. I do not pretend to say that this is always the case.

On the treatment I shall not say many words, only that emetics, purges, solution of sulphate of zinc, applied externally, and tonic medicines, are all very useful. In warm climates, where I have seen the complaint, I have known it easily cured, in a very short time, by confining the patient in the shade.

*Portsmouth; Oct.* 4, 1816.

